# Breast MRI: an illustration of benign findings

**DOI:** 10.1259/bjr.20220280

**Published:** 2022-12-09

**Authors:** Lyn Isobel Jones, Katherine Klimczak, Rebecca Geach

**Affiliations:** 1 Bristol Breast Care Centre, North Bristol NHS Trust, Bristol, United Kingdom

## Abstract

Despite its unparalleled sensitivity for aggressive breast cancer, breast MRI continually excites criticism for a specificity that lags behind that of modern mammographic techniques. Radiologists reporting breast MRI need to recognise the range of benign appearances on breast MRI to avoid unnecessary biopsy. This review summarises the reported diagnostic accuracy of breast MRI with particular attention to the technique’s specificity, provides a referenced reporting strategy and discusses factors that compromise diagnostic confidence. We then present a pictorial review of benign findings on breast MRI. Enhancing radiological skills to discriminate malignant from benign findings will minimise false positive biopsies, enabling optimal use of multiparametric breast MRI for the benefit of screening clients and breast cancer patients.

## Introduction

Multiparametric breast MRI is an accurate and important tool for the detection, diagnosis, characterization, and local staging of breast cancer.^1^ In clinical practice, it enables informed decision-making for the breast cancer patient and multidisciplinary team. The dynamic gadolinium contrast enhanced series of *T*1W scans at the heart of the multiparametric breast MRI protocol is the most sensitive method available for breast cancer detection^2–6^ and forms the basis of abbreviated protocols used for breast cancer screening.^7–9^ Additional sequences, including diffusion weighted imaging and T2W sequences, enhance lesion characterization.

The use of breast MRI to screen women at high risk of breast cancer, has been shown to reduce the incidence of late stage disease,^10,11^ and thereby increase metastasis-free survival^12^ when compared with mammography alone. These results are achieved through the ability of MRI to highlight small, aggressive cancers, including triple negative cancers, before they can be detected on a mammogram, spread to regional nodes, or become palpable clinically.

However, the success of breast MRI depends on skilled interpretation to optimize specificity and minimize biopsy rates. A breast Radiologist therefore needs an awareness of the MRI appearances of benign findings.

This article will present a short literature review of the diagnostic accuracy of multiparametric breast MRI, focusing on specificity. A systematic approach to MRI reporting is best practice^13^ so we include sections on reporting strategy and factors that affect diagnostic accuracy. A pictorial review is included of some benign pathologies encountered on breast MRI. Some of these findings will require biopsy to exclude cancer, but some are pathognomonic of a benign lesion and can avoid biopsy.

## Diagnostic accuracy of breast MRI with a focus on specificity and false positive biopsies

Meta-analyses of screening trials of breast MRI for BRCA carriers^14^ and for women at high familial risk without a known mutation^15^ report that although MRI’s sensitivity for breast cancer far exceeds that of mammography (85–89% v. 40–55%), its specificity remains inferior to that of mammography (83–85% v. 93–96%).

The specificity of a test is particularly important in breast screening, where large numbers of women are being tested for a condition that is present in only a small number (low pre-test probability). If there is a low pre-test probability, a small reduction in specificity can translate into many women without cancer being recalled and potentially having a benign biopsy.

The percentage of biopsies indicated by a test that turn out to be cancer is known as the positive predictive value for biopsy (PPV_3_) and, in any given population, is a useful measure of a screening test’s balance of benefit to harm. However, it is less useful for comparisons between different populations because PPV_3_ changes dependent on the pre-test probability. For the same test, the higher the pre-test probability of the population tested, the higher the PPV_3_. For example reported PPV_3_ is higher when the breast MRI is for local staging of cancer (PPV_3_ of contra-lateral breast 31%^16^ and of either breast 44%^17^ and 31%^18^) than for a screening context. In breast screening, PPV_3_ has been reported for MRI in USA as 21%^19^ and 24%^20^ and for BRCA carriers in Europe as 25%.^4^ For very high risk women within NHSBSP^21–23^ although the PPV for recall to assessment was 14%, the PPV_3_ for biopsy was 25%^24^ implying that 4 women were biopsied to find one cancer.

Interestingly, for mammographic-screening of population-risk women within NHSBSP, PPV_3_ is much higher for women at their second or subsequent screening round (incident round) (43–52%) than for women at first screen (prevalent round)(13–19%).^25^ Mammography’s higher specificity at incident screen is due to the availability of previous films for comparison, which also tends to enable increased cancer detection.^25^ In prospective studies and trials of MRI screening the reported increase in PPV_3_ in the incident round is less marked^2–5,15,26^ but it occurs despite a reduction in cancer detection rate at the incident round for MRI^2–5,26^ that, on its own, would have been expected to reduce PPV_3_. This demonstrates that access to previous MRI images helps radiologists to reduce false positive biopsies.

The reduction in cancer detection rate seen in the incident round is because breast MRI’s high sensitivity for biologically significant cancers at the prevalent round reduces the pre-test probability for women going into the incident round. MRI has already detected (and removed) all or most of the biologically significant cancers at the prevalent round, leaving for detection only those that have newly appeared during the screening interval.

This reasoning is supported by the following evidence. Cancers detected at breast MRI incident round are generally smaller and at an earlier stage than cancers detected at mammographic incident round. Specifically, the proportion of invasive cancers detected at MRI incident round that are <2 cm diameter has been reported as 13/14 (93%),^2^ 11/11 (100%),^3^ and 14/14 (100%).^26^ In comparison, only 55% of cancers detected by mammography at incident round are <1.5 cm (29943/54,068).^27^ A similar pattern is seen for the proportion of incident round invasive cancers that are node negative for MRI: 10/11 91%,^3^ 13/14 93%^2^ and 14/14 100%,^26^ compared with incident round screening mammography: 75–80% node negative.^28^ It is also supported by evidence that, for high risk women, screening with breast MRI reduces the incidence of late stage disease.^10,11^

For radiologists reporting breast MRI, optimizing specificity in pursuit of that achieved by mammography^14,15^ is key to maximizing the benefit to harm ratio for breast MRI use and making the most of its unparalleled sensitivity for aggressive cancer in both symptomatic and screening practice. Illustrating what could be attainable, several MRI screening studies published in the last decade have reported breast MRI specificity at incident round as above 90%: 91.8%,^2^ 98.4%,^3^ 96.2%,^4^ 96.5%,^5^ 98%.^26^

## Strategy for interpretation of masses, non-mass enhancement and foci of enhancement

The Breast Imaging – Reporting and Data System (BI-RADS) of the American College of Radiologists provides a widely accepted lexicon and reporting system.^29^ For breast MRI, BI-RADS classifies enhancing findings within the breast into masses, non-mass enhancement, foci of enhancement, and normal structures such as intramammary lymph nodes and vessels.

Some imaging features are more common in benign than malignant pathology.

For all enhancing findings, in multiparametric breast MRI, it is useful to perform simultaneous slice matched comparison of the different sequences (T2W, DWI, ADC map and both raw and subtracted, early and late dynamic images). This enables full appreciation of the characteristics of the enhancing tissue.

This article aims to describe benign appearances but if a mixture of benign and malignant features are present then biopsy is necessary to exclude malignancy. Appreciation of the findings that require biopsy is more useful to the reporting radiologist than a list of features that are usually benign.

Each category of enhancing finding has a different set of indications for biopsy:

### Enhancing masses

For enhancing masses, markers of malignancy include^30–32^ :

- a poorly circumscribed margin,
- rim or heterogeneous pattern of internal enhancement,
- a fast initial enhancement phase,
- either a plateau or a delayed phase washout curve,
- low apparent diffusion coefficient (ADC) values on diffusion weighted imaging (DWI)^33^
- size increasing on sequential scans

#### Diffusion-weighted imaging (DWI)

Through its appreciation of tissue microstructure and cellularity, DWI can improve breast MRI specificity for masses and thereby reduce the number of unnecessary biopsies.^34^ The EUSOBI working group published recommendations for its use in 2019, including the need for standardisation and quality assurance prior to setting Apparent Diffusion Coefficient (ADC) thresholds and the advice that ADC measurement should be made using a small region of interest (ROI) on the ADC map, placed on the darkest part of the lesion in question.^35^

The use of DWI in routine high risk screening practice has been credited with enabling screening centers to keep recall rates within the expected standard of <7%,^36^ a standard which has previously been reported as difficult to meet.^24^

#### Diagnostic keys

To simplify breast MRI reporting, several articles have suggested a diagnostic key, such as the Kaiser score (KS),^37^ to aid categorization of breast masses. Evidence from these studies supports the avoidance of biopsy for a mass (exclusion of malignancy) if it has both a circumscribed margin and a slow, persistent enhancement curve.^38,39^

A recently published comparison of the use of DWI and KS to optimize breast MRI specificity suggested that KS achieved better discrimination between benign and malignant masses that DWI, especially for small masses < 1 cm diameter.^40^

### Non-mass enhancement

Malignant lesions presenting as non-mass enhancement (NME) often exhibit benign kinetic patterns of enhancement.^32^ The only two discriminators between benign and malignant NME available to the radiologist are the distribution and internal enhancement pattern.

For NME, the highest predictors of malignancy are:

- segmental, asymmetric distribution
- clustered ring or a clumped enhancement pattern.^41^

Malignant NME can have:

- any type of initial enhancement phase including “slow”,
- any type of late enhancement phase including “persistent”,^32,33^
- any appearance on DWI including with a high ADC^33^ and
- malignant NME may not change perceptibly on sequential scans.^32^

### Enhancing foci

Enhancing foci are, by definition, too small to discern margin detail so the morphology cannot be assessed. Unlike masses, foci with a high *T*_2_-weighted signal or a high ADC value on DWI cannot necessarily be considered benign. However, they have the lowest PPV_3_ of the three categories of enhancing findings on MRI.^16,18^

A single focus of enhancement in an otherwise minimally enhancing breast should be assessed kinetically, since a higher PPV_3_ is seen for foci with

- a fast initial phase^42^ and/or
- a washout delayed phase.^43^

Particular care must be taken when assessing the time intensity curve for a focus since, because of its small size, motion artifact can distort the curve and falsely simulate a washout pattern. A careful check for motion artifact and correlation with source data are both essential to avoid unnecessary recall.

To determine if a focus of enhancement is truly solitary it is best assessed on the first post-contrast images of the dynamic sequence to maximise the difference in enhancement between lesion and background^32^ as malignant foci enhance earlier than background parenchymal enhancement.^42^

Multiple foci, especially if bilateral, are extremely likely to represent benign parenchymal enhancement (BPE). The exception is if the distribution of the foci is asymmetric and segmental or regional when they can then collectively be treated as NME that requires biopsy. For asymmetric regional non-mass enhancement >10 mm, the PPV is greater than 25%.^44,45^

## Factors affecting diagnostic accuracy of multiparametric breast MRI

Factors that may affect the diagnostic accuracy of breast MRI include background parenchymal enhancement (BPE) and motion artifact. However, appreciation of the typical features of each minimizes this effect for experienced readers.^46^

### Background Parenchymal Enhancement (BPE)

Moderate or marked levels of BPE lead to increased recall rates and biopsy without increased cancer detection.^47^ However, high levels of BPE are associated with increased risk of developing breast cancer. Women with a high familial risk of breast cancer who also have moderate and marked BPE are significantly more likely to develop breast cancer in the next 5 years than those with minimal or mild BPE.^48^ It is important that BPE is minimized through optimization of the timing of MRI examination within the menstrual cycle, for example, by performing screening scans at day 6–16 and repeating the MRI at 6 weeks if moderate or marked BPE is shown.^23^

Appreciation of the different patterns that are typical of BPE is important to optimize specificity. The distribution of BPE is typically symmetrical and includes the following patterns^49^ :

- around the periphery of the fibroglandular parenchyma (“picture framing” or “cortical”)
- central
- diffuse foci.

### Movement artifact

Recognising movement artefact and understanding how it can cause pseudo-enhancement on subtracted images is important to prevent unnecessary false positives.^50^ Motion artefact has characteristic curvilinear paired dark and light markings that outline the pattern of the underlining normal parenchymal tissues.

### The post-conservation and reconstructed breast

Following cancer diagnosis, both breast conservation and post-mastectomy reconstruction bring an additional a set of benign appearances on MRI for the radiologist to appreciate.

**Figure 1.**
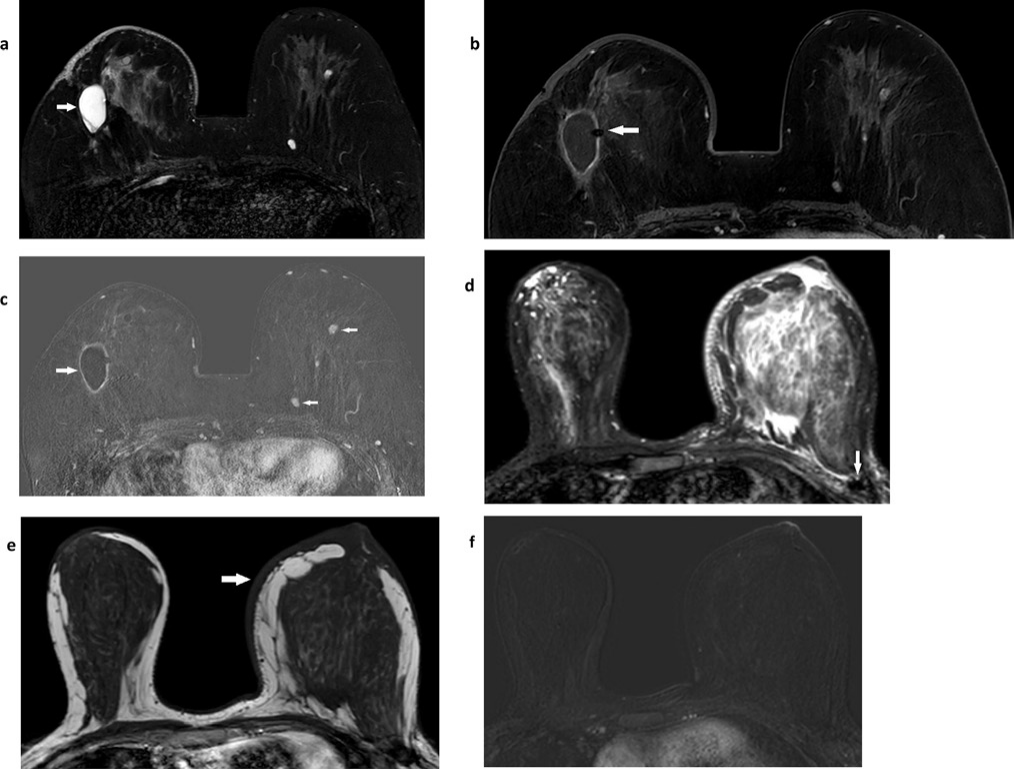
Breast conservation appearances Two cases illustrate breast conservation post-radiotherapy appearances. Case 1: one year after right breast conservation surgery and radiotherapy, there is a seroma cavity (white arrow) that is high signal on fat suppressed T2W (**a**) with some low signal stranding within the seroma cavity. Skin thickening and edema of the skin and within the breast are also demonstrated. Fat suppressed T1W post-contrast from the dynamic series (**b**) demonstrates a thin, uniform rim of enhancement with a signal void from a surgical clip (white arrow) in the seroma’s medial border. The equivalent subtracted image (**c**) confirms enhancement in the seroma’s rim and in two small ovoid masses with benign morphology (white arrows) in the contralateral, left breast which have the typical appearance of fibroadenomas. Case 2: one year after left breast conservation and radiotherapy. There is marked skin thickening (white arrow) and oedema with resultant swelling of the conserved, left breast illustrated on fat-suppressed T1W (**d**), T2W (**e**) and a subtracted post-contrast image from the dynamic series that shows no abnormal enhancement (**f**). The surgical site is in the far lateral left breast, seen as very low signal scar tissue on both T1W and T2W images (white arrow).

A seroma cavity filled with fluid, that may contain blood and have a thin enhancing rim, is a common finding on breast MRI in the first year after surgery and can persist beyond 5 years^51^ (Figure 1).

In the case of post-operative histologically positive margins, breast MRI is sometimes performed to help decide between re-excision of margins and mastectomy. Assessment is made for any suspicious areas of enhancement large enough or remote enough from the cavity that would not be removed by a standard re-excision of margins.

Since it is not possible to reliably identify or quantify residual low to intermediate grade DCIS, especially in the presence of post-surgical inflammatory change, managing surgical expectations of post-surgical MRI is important because a negative MRI in this context will not obviate the need for re-excision of margins and a positive MRI will not necessarily require mastectomy if enhancement is present solely within the region of the planned margin re-excision.

Radiotherapy following breast conservation surgery for cancer treatment commonly causes skin thickening and oedema (seen on T2W images). These benign changes persist for more than 5 years in a quarter of cases^51^ (Figure 1).

**Figure 2.**
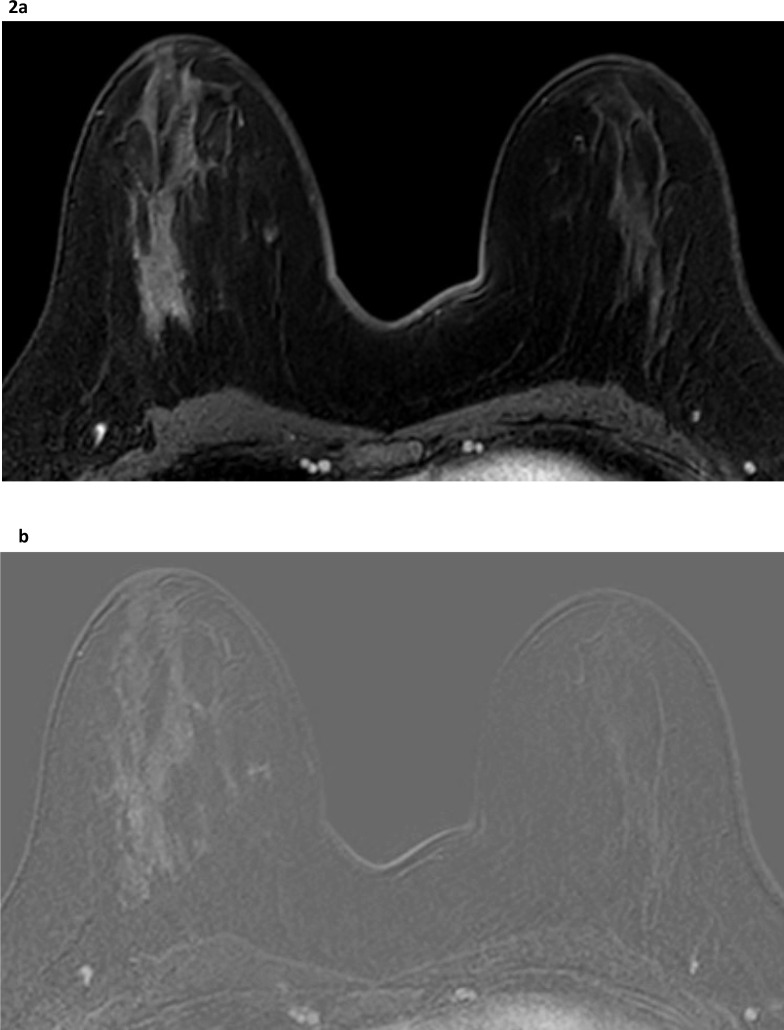
Asymmetric enhancement post radiotherapy. Three years after left breast conservation surgery and radiotherapy, a slice from a post-contrast sequence of the dynamic series (**a**) demonstrates asymmetric enhancement of the fibroglandular tissue with reduced enhancement in the irradiated left breast. The asymmetric nature and distribution of background parenchymal enhancement in the contralateral, right breast is normal and should not be confused with asymmetric, abnormal non-mass enhancement. The equivalent subtracted image (**b**) confirms the enhancement in the normal fibroglandular structures of the right breast and its asymmetry in comparison with the left.

Radiotherapy also has the effect of reducing background parenchymal enhancement, and although this effect has been demonstrated bilaterally,^51^ it is more marked in the conserved breast and results in an asymmetric appearance (Figure 2). If unaware of the clinical history, the reporting Radiologist may confuse the asymmetric normal BPE in the contralateral breast with abnormal non-mass enhancement, leading to a false-positive MRI report.

**Figure 3.**
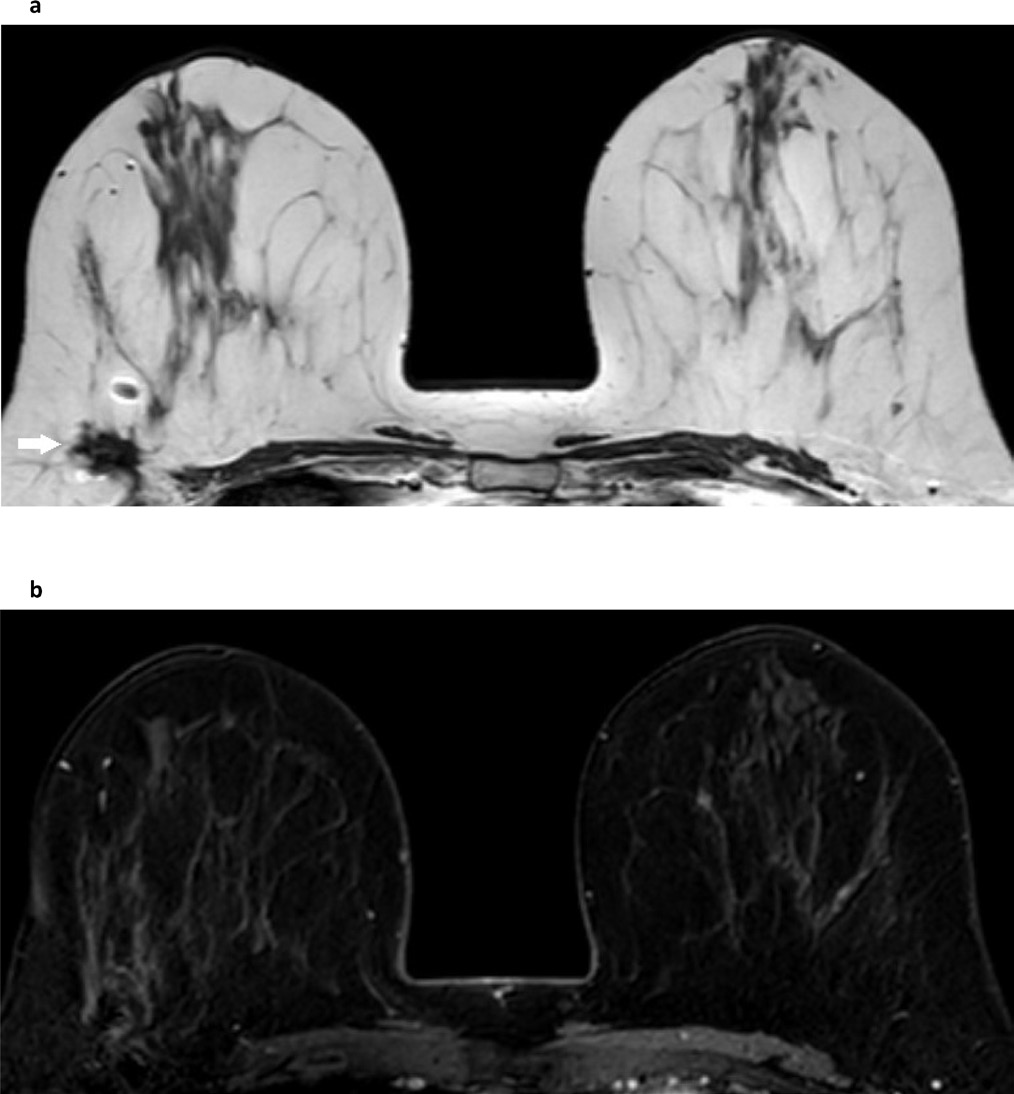
Breast conservation enhancement in the scar. One year after surgical conservation of the right breast non-fat-suppressed T1W (**a**) shows a low signal fibrous scar (white arrow) laterally that contrasts with the surrounding fat, and a rectangular signal void from a surgical clip anteromedial to it. **b** is the same slice from the dynamic sequence post contrast, demonstrating some subtle asymmetric enhancement surrounding the fibrous scar. This is a common post-surgical finding at one year.

Surgical clips and scarring within a breast are more likely to exhibit motion artefact on subtracted images than other parts of a conserved breast, and can have the appearance of pseudo-enhancement. Surgical scars are fibrotic and tend to have low signal on T1W and T2W, but in the first year after surgery the surgical site commonly exhibits asymmetric enhancement^51^ (Figure 3).

Acellular dermal matrix (ADM) is a type of surgical mesh which can be implanted as a sheet or cut into cubes. It is being increasingly used in both breast conservation surgery and breast implant reconstruction to improve cosmetic outcomes and reduce infection rates. On MRI, the ADM may be iso or hypointense to fibro-glandular tissue on T1W and T2W, but crucially it does not enhance and can therefore be recognised as a benign finding.^52,53^

**Figure 4.**
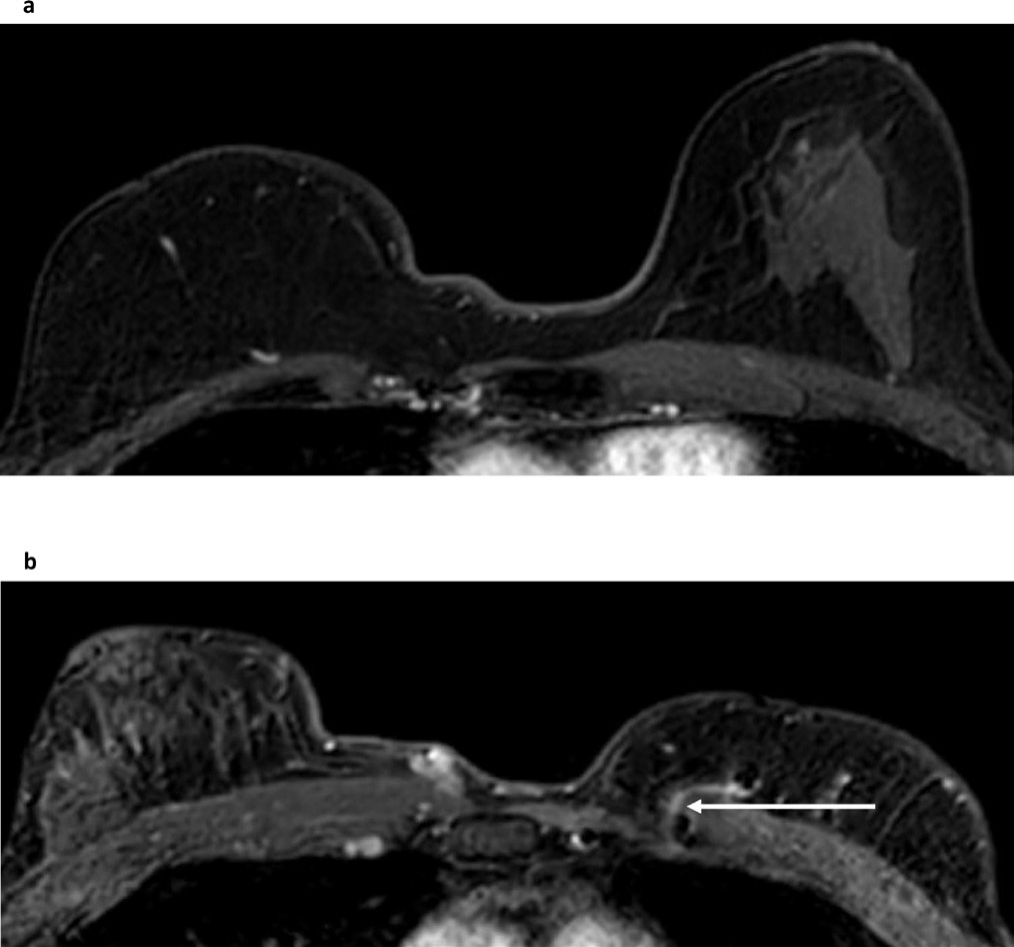
Deep inferior epigastric perforator (DIEP) free flap reconstruction. Two cases illustrate the appearances on breast MRI of free-flap reconstruction with a deep inferior epigastric perforator flap (DIEP). Both are illustrated with a post contrast fat-suppressed T1W image from the dynamic series, and both show the typical broader and flatter appearance of a DIEP-reconstructed breast on MRI. Case 1 demonstrates the asymmetric lack of fibroglandular tissue in the DIEP-reconstructed right breast (**a**). Case 2 shows the anastomosis of the host internal mammary vessels to the perforator vessels (white arrow), seen medially in the DIEP-reconstructed left breast (**b**).

Post mastectomy reconstruction options include synthetic implants with or without ADM, autologous pedicled flap (for example using latissimus dorsi) and autologous free flap reconstructions. Knowledge of the expected configuration of tissue and resultant appearances on MRI of the common reconstruction options, including an awareness of the site of the vascular pedicle is essential to recognize the spectrum of benign appearances of the reconstructed breast^52^ (Figure 4).

Surgery can be considered a type of trauma and therefore, fat necrosis is a common finding in the post-surgical breast. It is a particularly common finding after pedicled transversus abdominus muscle (TRAM) flap reconstruction,^54^ but has also been reported in 18% of free flap autologous breast reconstructions. In the deep inferior epigastric perforator (DIEP) flap),^55^ fat necrosis is most likely to occur in the lateral part of the reconstructed breast which is furthest from its new blood supply.^52^

## Benign diagnoses

### Fibrocystic change

Fibrocystic change is the most common benign disorder of the breast. It most frequently affects pre-menopausal women.^56^ Fibrocystic change includes a broad spectrum of histological findings. The spectrum comprises both non-proliferative and proliferative conditions ranging from cysts and duct ectasia, through radial scar, adenosis and papilloma to epithelial hyperplasia with or without atypia.^56,57^

**Figure 5.**
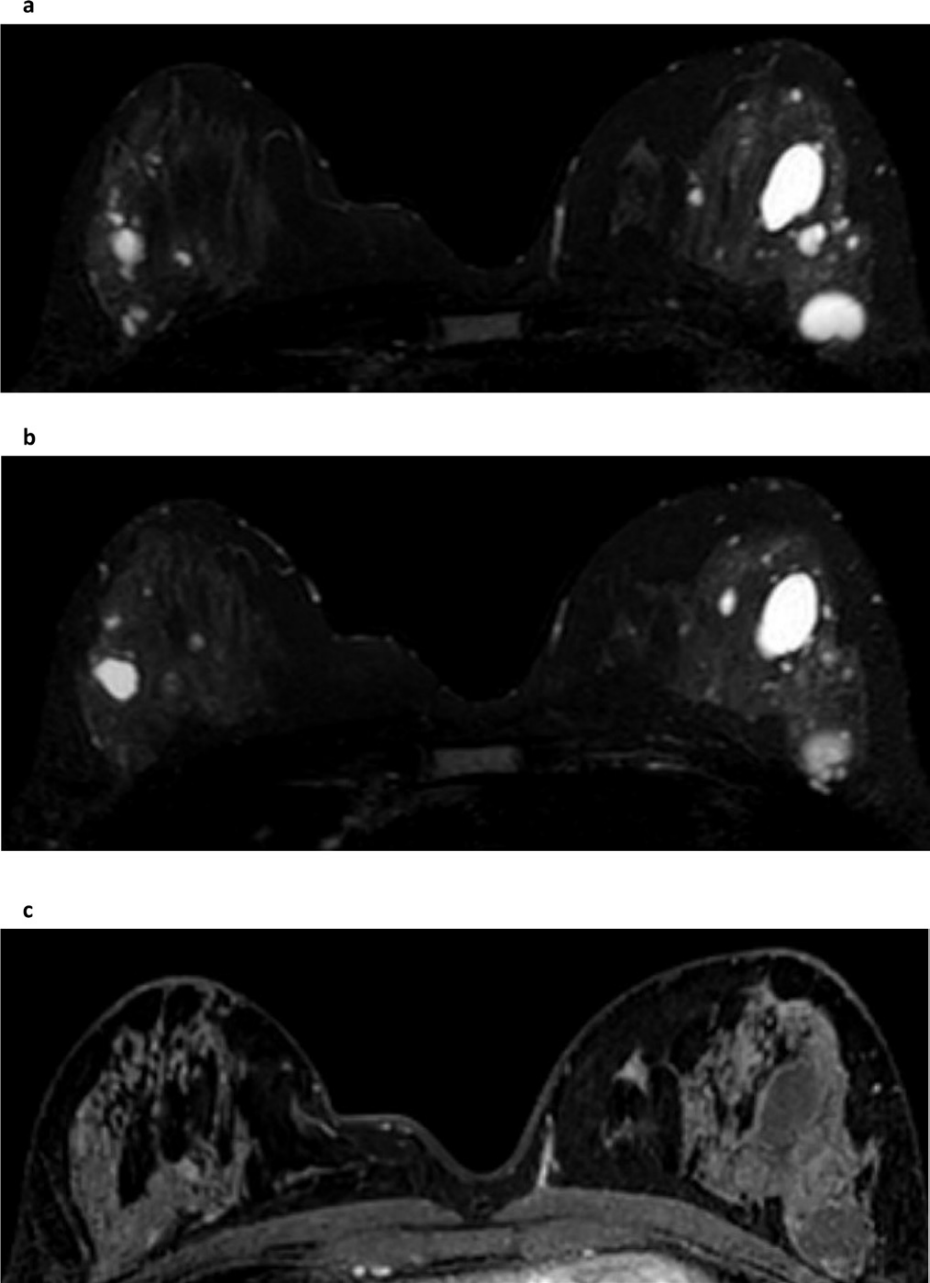
Simple cysts. Two slices from the T2W sequence stack of images (**a** and **b**) demonstrate multiple and bilateral simple cysts with thin walls that on fat-supressed T1W dynamic sequence (**c**) do not enhance and are slightly hypo-intense to fibroglandular tissue but hyper-intense to fat.

Simple cysts are a common incidental finding on breast MRI. Cysts are well circumscribed lesions and are typically multiple and bilateral (Figure 5). Since they are fluid filled structures, in all sequences their contents typically exhibit uniform fluid signal (high signal on T2W and low signal on fat suppressed T1W). Their key feature is that they do not enhance, and this is best appreciated on the subtraction sequences.

**Figure 6.**
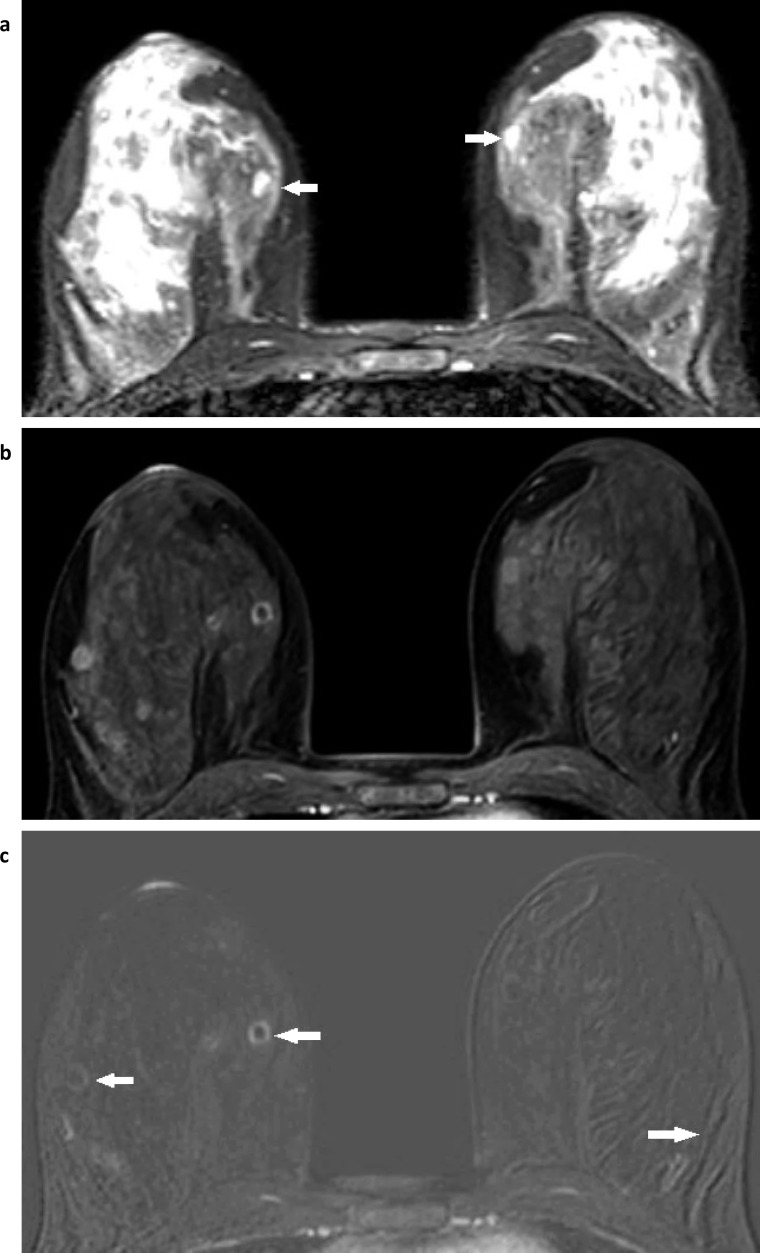
Cyst with enhancing wall. Small cysts are seen medially in the breasts bilaterally (white arrows) on this fat-suppressed T2W image (**a**) and there is also high signal within the fibroglandular tissue. A fat-suppressed T1W dynamic post contrast slice (**b**) shows enhancement of the cyst wall in the medial right breast and slight hyperintensity of signal in several small cysts laterally in the right breast and in one cyst medially in the left breast. The equivalent subtracted slice (**c**) demonstrates that the only enhancement is in the walls of two cysts, one in the medial right breast, and also more subtle enhancement in the wall of a second small cyst in the lateral right breast (white arrows). Some movement artefact is noted incidentally at the back of the far lateral left breast (white arrow), seen as paired curvilinear dark and light lines that follow the contours of the underlying fibroglandular tissue.

Complicated cysts may have septations, fluid/fluid levels or debris from internal protein or haemorrhage that result in cyst contents having low signal intensity on T2W and low ADC values. Cyst walls may enhance if there is inflammation present, but the cyst wall must be uniformly thin to allow confident diagnosis on MRI without biopsy^58^ (Figure 6). Again, review of the dynamic and subtraction series is essential to characterise any enhancement present.

**Figure 7.**
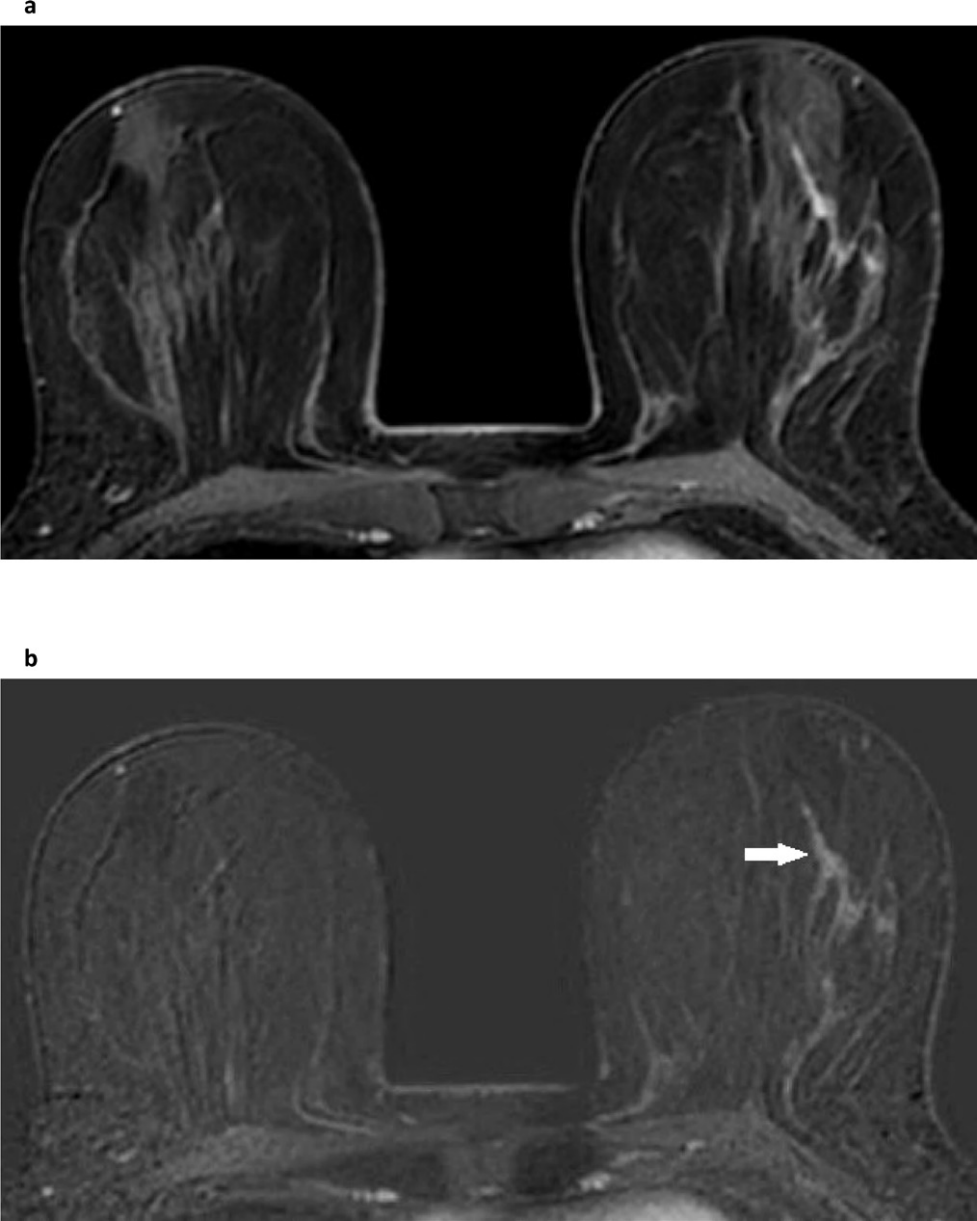
Fibrocystic change as non-mass enhancement. Late post contrast fat-suppressed T1W image from the dynamic series (**a**) and equivalent subtracted image (**b**) from the MRI of a high-risk screening client with Li Fraumini syndrome, showing new linear non-mass enhancement (white arrow) in the lateral left breast. MRI-guided biopsy showed that this was fibrocystic change with periductal mastitis.

A range of diagnoses can appear as complicated or complex cysts on MRI. These include galactocoele, hematoma, fat necrosis, oil cyst, breast abscess, intracystic papilloma and necrotizing malignancy.^59^ Non-enhancing, complicated cysts have only a 0.2% chance of being malignant.^59^ However, if a cyst has an irregularly thickened enhancing wall or contains an enhancing mass, biopsy is essential to exclude malignancy. A region of fibrocystic change can appear as non-mass enhancement and if asymmetric can require a biopsy for confirmation (Figure 7).

### Duct ectasia

Duct ectasia is a frequent incidental finding on breast MRI. Dilated ducts are seen predominantly in the subareolar region with fluid contents that are high signal on T2W. Importantly, the contents of the ducts are often also high signal on the T1W with fat saturation sequence, used for the dynamic series, because of proteinaceous content. Therefore, in assessing duct ectasia, it is essential to review the subtracted images, because duct ectasia does not enhance and enhancement in this context could represent papilloma or ductal carcinoma *in situ* (DCIS).

### Fibroadenoma

Fibroadenoma is the commonest benign breast mass in women and is discussed separately within the current issue of this journal, so will be dealt with only briefly here.

Fibroadenoma is the most common single benign lesion identified at image-guided biopsy of suspicious findings at breast MRI (quoted variously in recent studies as 21–52% of such biopsies^33,38,60^). A fibroadenoma is a benign tumour of mixed epithelial and stromal elements which has a diverse appearance on MRI reflecting variation in the proportion of fibrous and myxoid elements. Sclerosed fibroadenomas exhibit low signal on T2W images, whereas myxoid fibroadenomas have high signal on T2W.^61^ The enhancement exhibited by a fibroadenoma is varied and in general myxoid fibroadenomas enhance more intensely than others.^61^

On MRI, a fibroadenoma is typically ovoid or round with a well circumscribed margin. It may show dark internal septations; these are low signal on T2W, non-enhancing bands within an enhancing lesion. The presence of septations has been reported as having a high specificity for fibroadenoma.^44^ This feature occurs in 30–60% of fibroadenomas but can also be present in Phyllodes (benign and malignant) and can be confused with some patterns of heterogenous enhancement seen in medullary carcinomas and mucinous carcinomas. Therefore, dark internal septations cannot be thought of as pathognomonic for fibroadenoma in isolation.^61–63^

**Figure 8.**
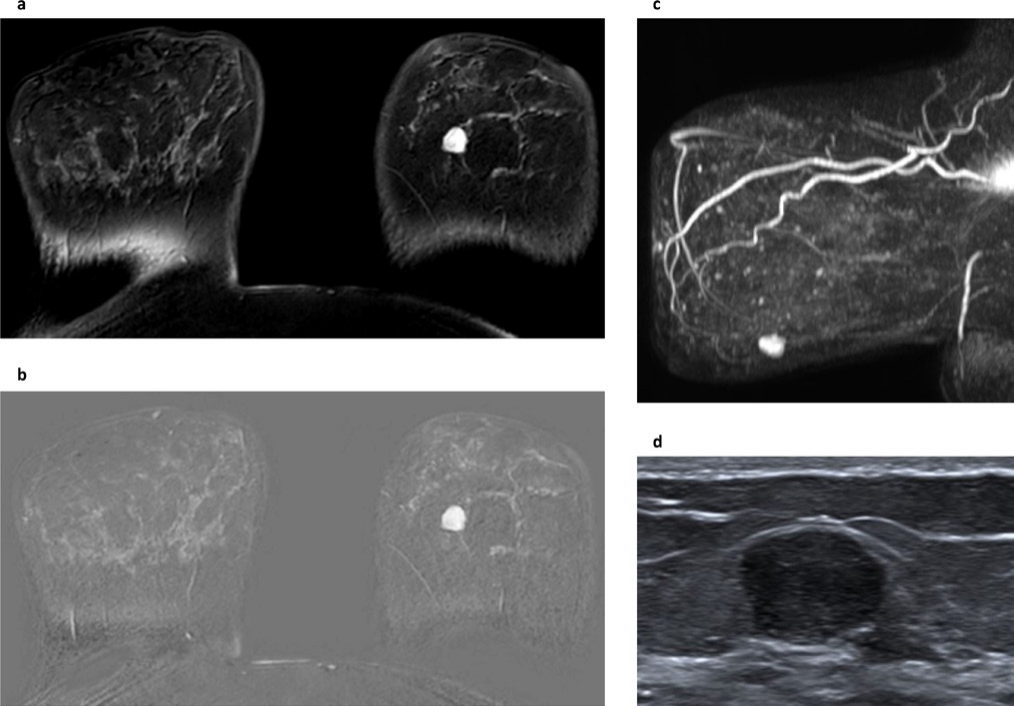
Fibroadenoma. This early post-contrast dynamic image (fat-suppressed T1W) (**a**) shows a biopsy-confirmed fibroadenoma in the left breast. It appears predominantly well circumscribed, but its high signal on this sequence represents avid enhancement which at this (early) time point demonstrates a fast initial enhancement curve. The equivalent subtracted image (**b**) contrasts the intense gadolinium uptake within the fibroadenoma with the much less intense normal bilateral background parenchymal enhancement and also shows that the internal enhancement pattern is heterogenous. **c** is the sagittal maximum intensity projection of the summated subtracted slices for the left breast and shows that the fibroadenoma is solitary and dominant within the breast. **d** is an image from the ultrasound correlation for the lesion.

A fibroadenoma may be clearly benign on breast MRI (Figure 1a–c). However, it can also have less clearly benign morphological features and a fast initial time intensity curve so that biopsy for diagnostic confirmation cannot be avoided (Figure 8). DWI can be helpful to avoid unnecessary biopsies of fibroadenomas as there is typically less restriction of diffusion than in malignant masses and therefore, even in the presence of a fast initial time intensity curve, a fibroadenoma’s ADC value may be sufficiently high to avoid biopsy.

### Phyllodes

Phyllodes are uncommon fibroepithelial lesions, accounting for less than 0.5% of breast tumours.^64^ Phyllodes may be classified as benign, borderline or malignant histologically, with benign phyllodes being the most common of the three types (approximately 40%).^65^

Typically, on MRI, phyllodes appear well circumscribed.

When compared with fibroadenomas, phyllodes are more likely to exhibit heterogeneity of internal enhancement and are less likely to have non-enhancing septations.^62^ Larger phyllodes with rapid growth may also exhibit high signal on T2W with internal cystic spaces and haemorrhage.^66^

### Papilloma

Intraductal papilloma is usually located centrally, within 3 cm of the nipple, and can be occult on MRI when small and benign.^67^ Larger papillary lesions appear on MRI most often as an enhancing mass (77%),^68^ which may be solid or mixed cystic/solid and have a variety of kinetic enhancement patterns,^69^ but can also be represented as linear non-mass enhancement.^70^ Associated duct dilatation is often visible.

### Abscess and infectious mastitis

Most commonly occurring in lactating women, infectious mastitis usually presents symptomatically with pain and redness, with a palpable mass and skin thickening on clinical examination. The differential diagnosis is inflammatory breast cancer, necessitating biopsy if unresponsive to antibiotics.

**Figure 9.**
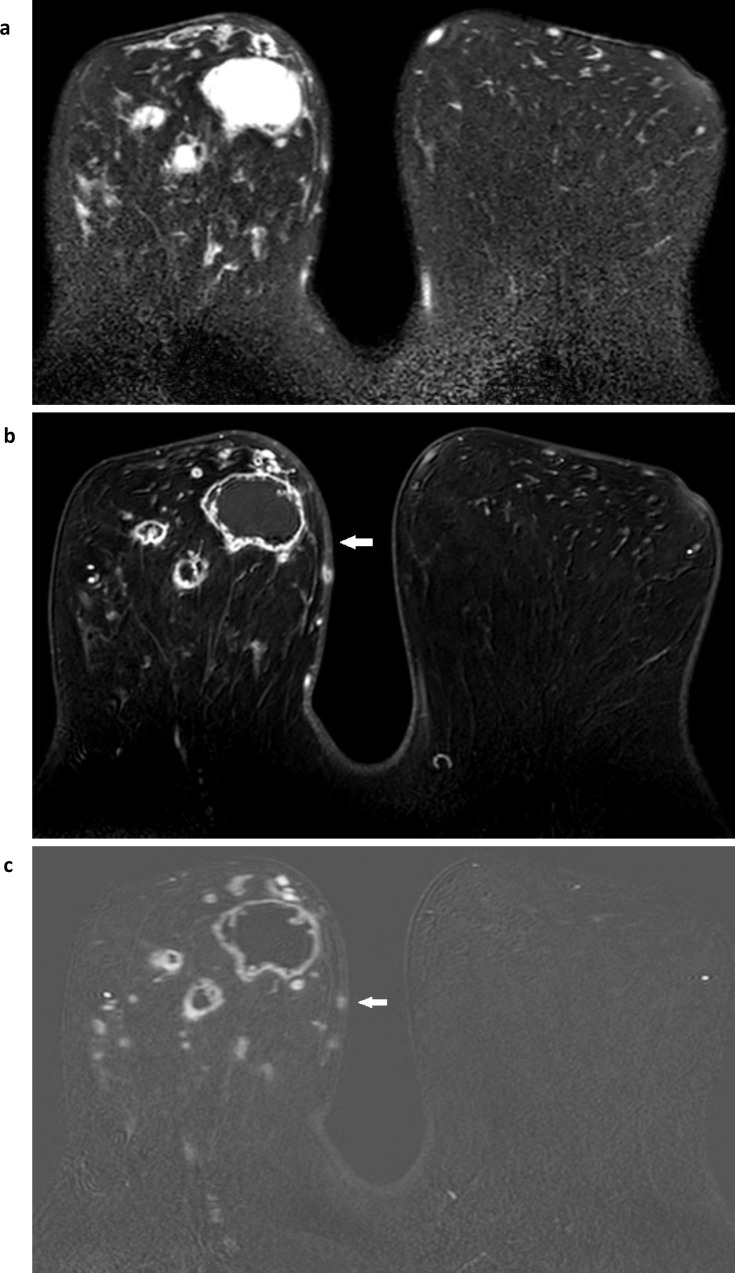
Breast abscesses. Multiple right breast abscesses: Fat suppressed T2W sequence (**a**) demonstrates multiple fluid-filled spaces in the right anterior breast with some background asymmetric oedema. Post-contrast from the dynamic series (fat-suppressed T1W) (**b**) better demonstrates the associated skin thickening of the medial right breast (white arrow) and shows the irregularly thickened and enhancing walls of the multiple right breast abscesses. The equivalent subtracted slice (**c**) confirms the irregular rim-enhancement in the abscesses and that there is only a small focal region of enhancement (white arrow) within the thickened skin of the medial right breast.

On MRI, an abscess is seen as an irregularly thickened, rim-enhancing mass with internal fluid signal (Figure 9). Over half of infectious mastitis cases have been shown to have a mass on MRI if imaged early in the infection.^71^ However, the commonest MRI appearance of infectious mastitis over the whole course of the infection is NME. This NME typically has a regional or multiregional distribution, often as clustered rings which probably represent micro-abscesses.^61^ Infectious mastitis is less likely to cause intramuscular pectoral edema than inflammatory carcinoma, and the location of enhancement in infective mastitis is typically in the subareolar region.^71^ Whilst this location favors an infective etiology over inflammatory cancer, it does not exclude DCIS as a differential. Clumped enhancement in the subareolar region requires a biopsy, especially in the absence of infective symptoms.

### Granulomatous mastitis

Granulomatous mastitis is a rare inflammatory condition that particularly affects women of child-bearing age who have breast fed, and is more common in women of Mediterranean, Asian and Arabic origin.^72^ The cause of the inflammation is unknown but hormonal, metabolic and traumatic aetiologies have all been proposed and the subtype cystic neutrophilic granulomatous mastitis is associated with the presence of Corynebacterium species.^73^

Imaging findings, including MRI appearances, are not specific and, since the differential diagnosis includes inflammatory carcinoma, image-guided biopsy is recommended to confirm the diagnosis. The histopathology is characterised by non-necrotic granulomas with multinucleated giant cell, histiocytic and lymphocytic infiltration.^72^ Small series of MRI findings have been described, which report multiple poorly circumscribed enhancing masses surrounded by NME. The masses exhibit a mixture of internal enhancement patterns and curves, including the development of abscesses with irregular, rim-enhancement morphology.^74,75^

### Fat necrosis

Fat necrosis is a benign, sterile, inflammatory process that can mimic cancer clinically and radiologically. It typically occurs after trauma, including breast surgery and following biopsy, radiotherapy, or breast infection. Fat necrosis can also be idiopathic.^76^ It has been estimated to account for 3% of biopsied breast lesions.^61^

**Figure 10.**
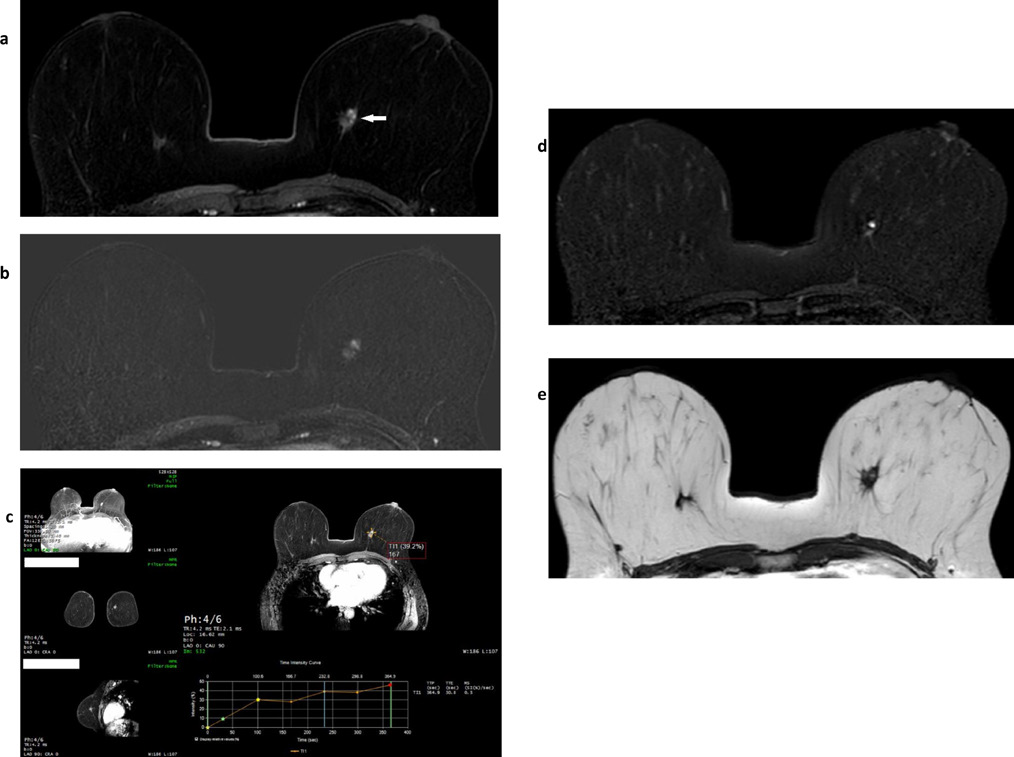
Fat necrosis. Post bilateral breast reduction, a poorly circumscribed mass (white arrow), with heterogenous internal enhancement, within the scar in the lower left breast is shown on a fat suppressed T1W image from the dynamic series (**a**) and on the equivalent subtracted image (**b**). Kinetically, it has a slowly rising persistent enhancement curve (**c**). It is also heterogenous with some high signal on fat suppressed T2W (**d**). However, on non-fat-suppressed T1W, although a partially spiculate morphology is demonstrated, there is high signal within the lesion that matches the signal intensity of the surrounding fat and enabled a diagnosis of fat necrosis within a scar to be made on MRI without biopsy (**e**). The symmetry of scarring from the breast reduction is also noted on this image.

Fat necrosis most commonly appears on breast MRI as a lipid cyst, sometimes with a fat/fluid level and a thin rim of enhancement. However, the appearance of fat necrosis on MRI varies with the amount of inflammatory component and is dependent on the presence of hemosiderin deposition.^77^ Crucially it can mimic cancer both morphologically and kinetically. The inflammatory component in fat necrosis can cause a fast rising washout curve and its fibrotic component can result in a poorly circumscribed or even spiculated margin morphologically. Avoiding biopsy may not be possible, but careful assessment of the lesion to see if the MRI signal at its center is similar to that of the adjacent fat on the unenhanced images can reassure and avoid biopsy, even in the presence of poorly circumscribed margin and rim enhancement^50,77^ (Figure 10). Additionally, correlation with the appearance on conventional imaging can also be helpful in making this diagnosis.

### Complex sclerosing lesion or radial scar

**Figure 11.**
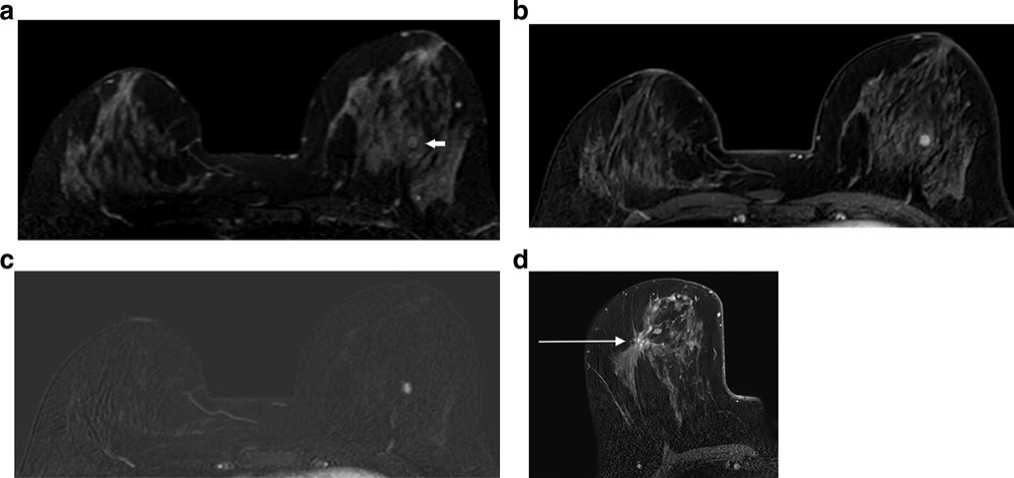
Radial scar. (**a**), (**b**) and (**c**) show a radial scar in the central left breast manifested on breast MRI as a small well-circumscribed enhancing mass (white arrow), that is barely visible on fat suppressed T2W imaging (**a**). The radial scar is clearly seen on a post contrast, fat suppressed T1W image from the dynamic series (**b**) and its enhancement is confirmed on the equivalent subtracted slice (**c**). (**d**) shows a different case with a typical appearance of a radial scar. The image is a T1W delayed high resolution image, kindly provided by Sarah Vinnicombe, MRCP FRCR, Consultant Radiologist, Cheltenham General Hospital, Gloucestershire Hospitals NHS Foundation Trust, and shows distortion with delayed enhancement within the central right breast (white arrow).

Up to 64% of radial scars and complex sclerosing lesions may be occult on MRI,^78–80^ but they can also appear as either a mass or NME. They can have a range of morphological and kinetic characteristics (examples of both atypical and typical appearances of radial scars are shown in Figure 11), including suspicious appearances that cannot avoid biopsy, such as non-circumscribed masses with heterogenous or rim enhancement.^78–80^

### Pseudoangiomatous stromal Hyperplasia (PASH)

PASH is a benign proliferation of fibrous stroma containing pseudovascular spaces lined by myofibroblasts that is believed to be hormonally responsive, occurring typically in premenopausal or perimenopausal women.^81^ Although it has been noted in up to 23% of breast biopsy specimens, it is relatively rare for PASH to present clinically as a palpable and enlarging mass.^61^

**Figure 12.**
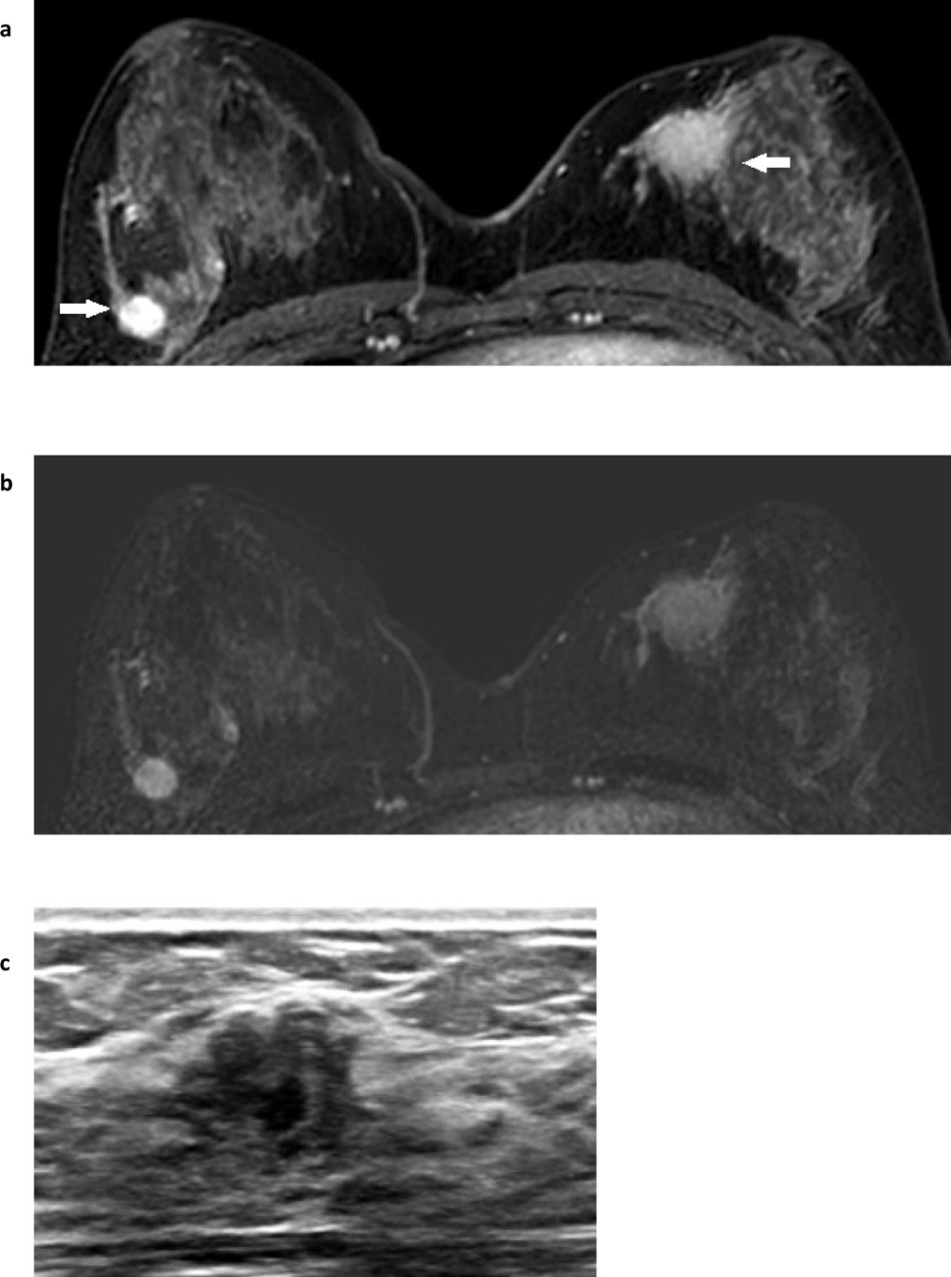
Pseudoangiomatous stromal hyperplasia (PASH). Post contrast (**a**) and subtracted (**b**) images from the dynamic series demonstrate a cancer in the medial left breast, and biopsy-confirmed pseudoangiomatous stromal hyperplasia (PASH) in the lateral right breast, both represented by enhancing masses (white arrows). The morphology and internal enhancement patterns are contrasted between the poorly circumscribed but homogenously enhancing cancer in the medial left breast and the better circumscribed margin of the right breast’s PASH that has heterogenous internal enhancement, presumably representing the slit-like cystic spaces within fibrous stroma that characterise PASH histologically. **c** illustrates an image from the second-look ultrasound that identified the impalpable PASH for biopsy and shows an irregular complex cyst with thickened hyper-echoic halo, cutting across tissue planes but with distal acoustic enhancement.

On MRI it can appear as a mass (Figure 12), but more typically shows as clumped NME in a variety of distributions^82^ and slit-like high signal cystic spaces within low signal fibrous stroma on T2W sequences.^70^ Although PASH usually has a persistent time intensity curve, biopsy is often indicated, especially to exclude DCIS.

#### Fibromatosis

Breast fibromatosis was defined by the World Health Organization as a locally infiltrative lesion without metastatic potential arising from fibroblasts or myofibroblasts.^65^ It is rare, accounting for less than 0.2% of breast tumors.^83^ One published series suggests it may occur more commonly in women who have had previous breast surgery.^84^ Breast MRI may be used:

- to define the extent of fibromatosis pre-operatively, since effective surgical treatment of symptomatic cases requires a clear margin^85^
- to monitor medical therapy, for example with tyrosine kinase inhibitors.^86^

**Figure 13.**
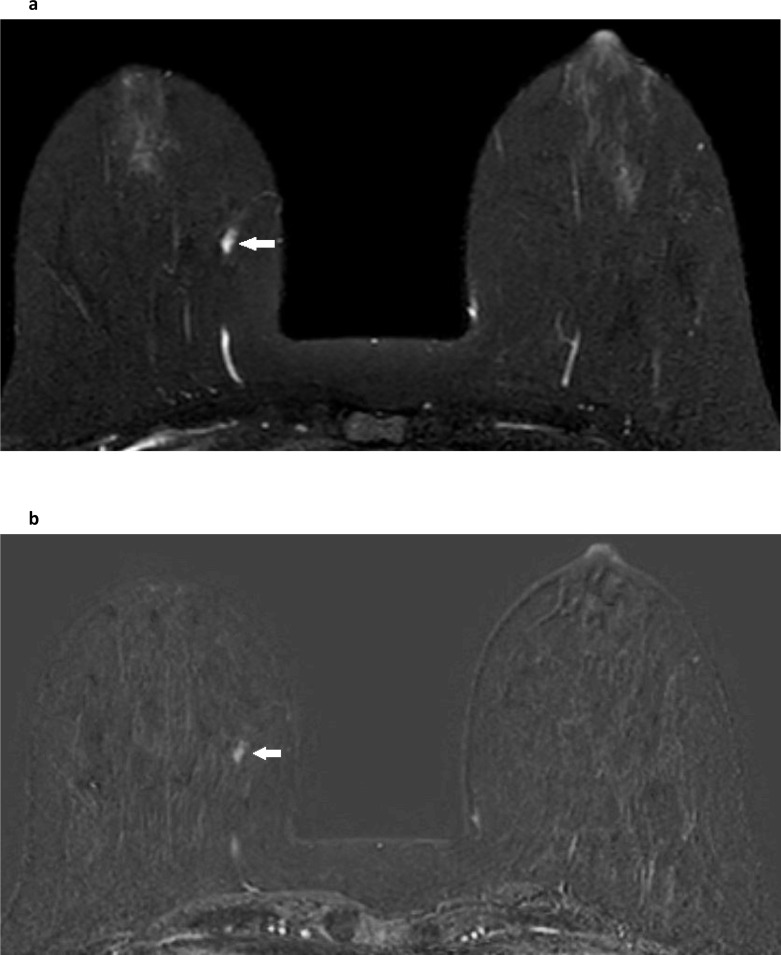
Fibromatosis. This breast MRI was performed for problem-solving following a symptomatic presentation of a suspicious-looking visible dent in the lower inner quadrant of the right breast. There was no palpable mass or other abnormality on examination and both mammography and ultrasound were normal. Fat-suppressed T2W imaging (**a**) showed a small hyperintense region (white arrow) in the medial right breast, and subtracted images from the dynamic series (**b**) showed a small focus of non-mass enhancement (white arrow). This was shown to represent fibromatosis at MRI-guided biopsy and at subsequent surgical excision.

Fibromatosis has most commonly been described as presenting as a palpable mass, and only a handful of descriptions of its appearance on MRI are found in the literature.^83,84,87^ It can have a variety of appearances on MRI, as a mass or as NME, iso or hypointense on T1W and high signal on T2W without diffusion restriction (Figure 13). Various time intensity curves have been described from slow persistent to fast plateau. As a mass it may be well circumscribed, poorly circumscribed, or even spiculate with architectural distortion, and so can mimic a cancer morphologically and dynamically. Biopsy is needed for diagnosis.

### Low flow vascular malformation or anomaly

Although low flow vascular malformations are the commonest peripheral vascular malformations, they occur rarely in the breast. These malformations are classified as lymphatic, venous or mixed lymphatic-venous.

Classical appearances on MRI are of a collection of adjacent fluid-filled cavities which are low signal on T1W and high signal on T2W, often with fluid/fluid levels. Venous malformations often contain phleboliths (better seen as calcification on mammogram than MRI) and exhibit late enhancement of the slow flowing venous channels on post contrast and late dynamic images. Macrocystic lymphatic malformations can also exhibit late enhancement, but in contrast to venous malformations it is the walls of the vascular spaces that enhance in lymphatic anomalies rather than the vascular spaces.^88^ The appearances of vascular malformations can be confused with the clustered ring or clumped enhancement of DCIS but may be sufficiently pathognomonic to avoid biopsy.

## Incidental findings outside the breast and axilla

**Figure 14.**
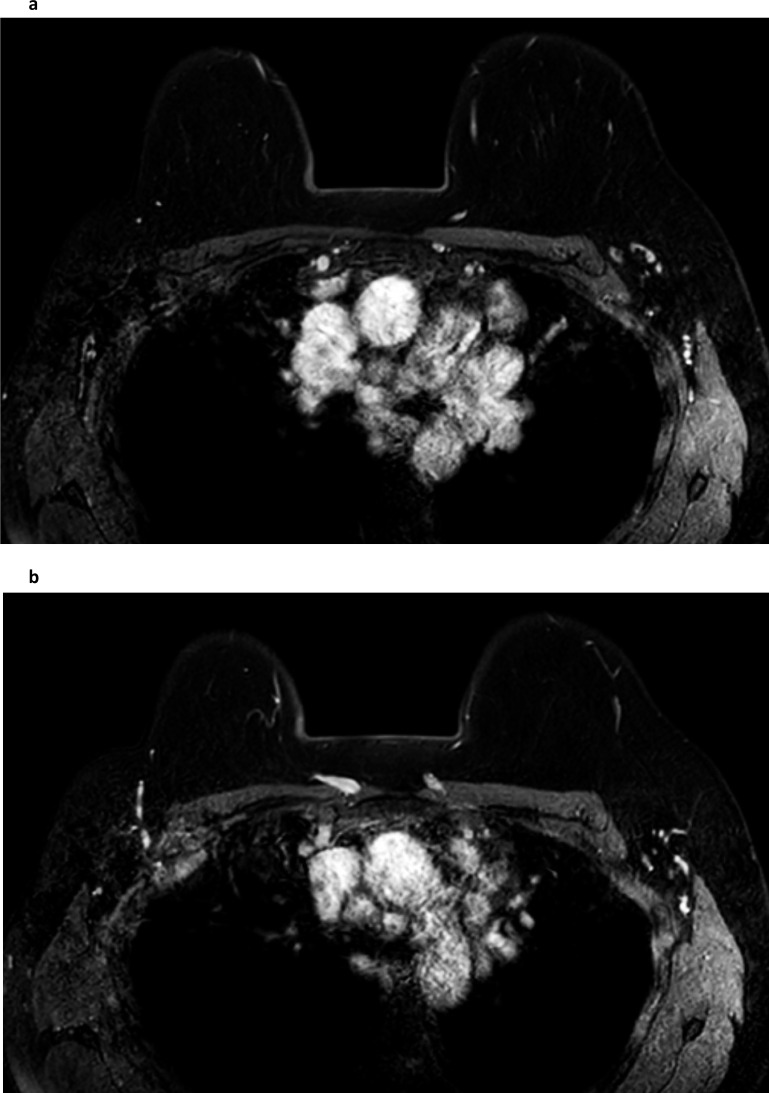
Incidental mediastinal lymphadenopathy. 4 years after a high-risk screening client had right breast conserving surgery and radiotherapy, her breast MRI demonstrated mediastinal lymphadenopathy, shown here on two slices from the post contrast dynamic series (**a** and **b**). Following investigation, a new diagnosis of sarcoid was made. These images are of the same patient as Figure 3.

Because the field of view of a breast MRI examination includes most of the thorax and part of the abdomen, especially with review of the scout images or localisers, it can identify findings outside the breast that are incidental to the indication for the study. Whilst approximately 20% have been reported as malignant, with the majority of malignant lesions being metastases in bone, lung, pleura, nodes or liver, the remaining 80% of incidental extramammary findings are benign^89,90^ (Figure 14). Nevertheless, it may be difficult to distinguish whether an incidental extramammary finding is benign or malignant on breast MRI and further imaging is often required.

**Figure 15.**
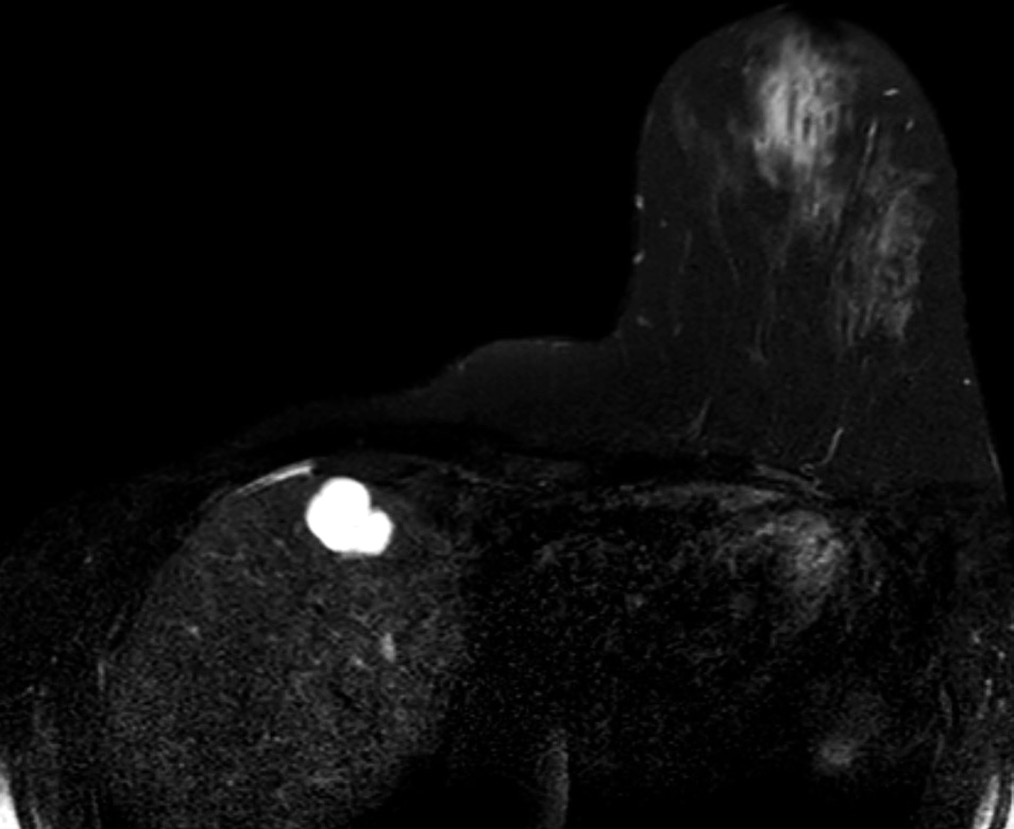
Liver cyst. This fat-suppressed T2W image shows a well circumscribed, lobulated, incidental liver cyst with septations in the right lobe of the liver of a patient who had previously undergone a right mastectomy.

Common benign extramammary findings seen on breast MRI include hepatic cysts (Figure 15) and hemangiomas, physiological pleural effusion, benign lung disease such as bronchiectasis, hiatus hernia and splenomegaly.^90^

Table 1 summarizes imaging characteristics by benign diagnosis.

**Table 1.** Summary of MRI imaging characteristics by benign diagnosis

Benign finding	Features	Signal	Enhancement	Additional information
**Seroma**	Well-defined collection at surgical site	Depends on blood/fluid content	No internal enhancement but can have a thin enhancing rim	Post-op can be present for over 5 years^50^
**Radiotherapy change**	Skin thickening edema	High signal on T2W	Reduced BPE	Asymmetric reduced BPE in breast exposed to radiotherapy – can cause unnecessary overcall of relatively increased BPE in contralateral breast
**Cysts**	Well-circumscribed lesions Round / oval Often multiple and bilateral	Uniform high signal on T2W Low on T1W fat sat	Either do not enhance or cyst wall enhances, if inflamed, but enhancing wall must be uniformly thin	Can have septations or fluid/fluid levels if proteinaceous
**Duct ectasia**	Dilated ducts in subareolar region	High on T2W High on T1W fat sat due to proteinaceous content	Does not enhance	Enhancement can indicate papilloma or DCIS so requires biopsy
**Fibroadenoma**	Ovoid / round mass Well-circumscribed margin	Varied signal: Can have low T2W internal bands / septations Sclerosed FA – low on T2W Myxoid FA – high on T2W On DWI – less restriction than malignant lesions	Non-enhancing internal bands within an enhancing lesion Myxoid types intensely enhancing	On DWI, uniformly high or very high ( ≥ 1.7 × 10 ^-3^ mm^2^/s^34^ ADC values within a mass can prevent biopsy for a morphologically typical fibroadenoma even if intensely enhancing
